# Tbit/s line-rate satellite feeder links enabled by coherent modulation and full-adaptive optics

**DOI:** 10.1038/s41377-023-01201-7

**Published:** 2023-06-20

**Authors:** Yannik Horst, Bertold Ian Bitachon, Laurenz Kulmer, Jannik Brun, Tobias Blatter, Jean-Marc Conan, Aurélie Montmerle-Bonnefois, Joseph Montri, Béatrice Sorrente, Caroline B. Lim, Nicolas Védrenne, Daniel Matter, Loann Pommarel, Benedikt Baeuerle, Juerg Leuthold

**Affiliations:** 1 ETH Zurich, Institute of Electromagnetic Fields (IEF), Gloriastrasse 35, 8092 Zürich, Switzerland; 2 ONERA, DOTA, Paris Saclay University, F-92322 Châtillon, France; 3 Thales Alenia Space in Switzerland, Schaffhauserstrasse 580, 8052 Zürich, Switzerland; 4 Polariton Technologies AG, 8803 Rüschlikon, Switzerland; 5 Present Address: Currently with LNE-SYRTE, Observatoire de Paris, Paris, France

**Keywords:** Fibre optics and optical communications, Adaptive optics, Atmospheric optics

## Abstract

Free-space optical (FSO) communication technologies constitute a solution to cope with the bandwidth demand of future satellite-ground networks. They may overcome the RF bottleneck and attain data rates in the order of Tbit/s with only a handful of ground stations. Here, we demonstrate single-carrier Tbit/s line-rate transmission over a free-space channel of 53.42 km between the Jungfraujoch mountain top (3700 m) in the Swiss Alps and the Zimmerwald Observatory (895 m) near the city of Bern, achieving net-rates of up to 0.94 Tbit/s. With this scenario a satellite-ground feeder link is mimicked under turbulent conditions. Despite adverse conditions high throughput was achieved by employing a full adaptive optics system to correct the distorted wavefront of the channel and by using polarization-multiplexed high-order complex modulation formats. It was found that adaptive optics does not distort the reception of coherent modulation formats. Also, we introduce constellation modulation – a new four-dimensional BPSK (4D-BPSK) modulation format as a technique to transmit high data rates under lowest SNR. This way we show 53 km FSO transmission of 13.3 Gbit/s and 210 Gbit/s with as little as 4.3 and 7.8 photons per bit, respectively, at a bit-error ratio of 1 ∙ 10^−3^. The experiments show that advanced coherent modulation coding in combination with full adaptive optical filtering are proper means to make next-generation Tbit/s satellite communications practical.

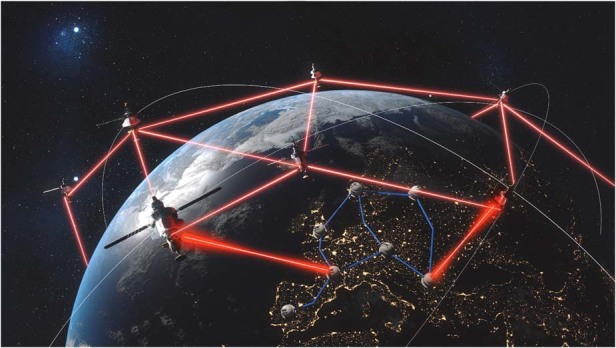

## Abstract (web summary)

This paper demonstrates a 53 km free-space-optical communication link mimicking a satellite-downlink. It achieves 1Tbit/s transmission by addressing turbulence and low-SNR issues through adaptive optics and a novel four-dimensional modulation format.

## Introduction

**Fig. 1:**
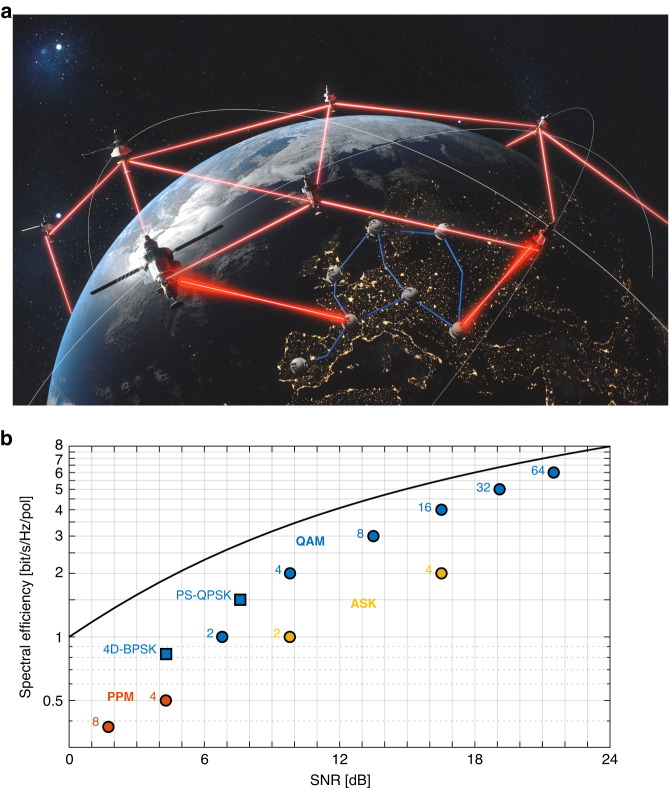
Optical space communication scenario and options for modulation formats. **a** Vision of optical satellite communication with satellite-earth links. Only a few ground-stations, interconnected with a continental fiber network, are needed to transmit and receive information to and from space. **b** Spectral efficiency [bits/s/Hz/pol] as a function of required signal-to-noise-ratio (SNR) for PPM (red), PAM (yellow), QAM (blue) modulation formats with bit-error rates of 1 ∙ 10^−3^ and as well as the required SNR according to the Shannon’s channel capacity theorem (plotted curve) as an upper limit. Also, two 4D QAM modulation formats (squares), namely polarization switched (PS) QPSK (PS-QPSK) and 4D-BPSK, are plotted that have recently shown highest sensitivities^44,51^. In comparison to polarization-multiplexed (PM) 4QAM, they have a sensitivity advantage of 0.97 dB and 1.7 dB respectively, at a bit-error ratio of 1 ∙ 10^−3^

Satellite constellation systems have the potential to provide global internet access from space, hereby connecting also “the unconnected”^1,2^, e.g. areas that do not rely on an existing infrastructure of alternative communication technologies such as optical fiber. Nowadays satellite communication links are based on RF communication, typically in the Ku-band (12–18 GHz) and Ka-band (26.5–40 GHz). By shifting the RF carrier frequencies to the optical frequencies, optical feeder links are perceived as a way to overcome the RF bottleneck and achieve high throughputs by exploiting the license-free vast optical bandwidth. Such optical links will then offer further scaling by wavelength-division multiplexing. In order to meet the link availability standards of satellite operators (usually 99.9%)^3,4^ around ten optical ground stations have to be integrated in a site-diverse network to bypass link blockages caused by clouds^5,6^, see Fig. 1a. Although RF technologies are less susceptible to the effects of clouds and other weather conditions on visibility^7–14^, the available RF bandwidth per ground station is typically limited to only a few GHz in the licensed spectrum. Consequently, they still require five times more ground gateways than optical technologies^15–17^ to achieve accumulated system throughputs in the order of Tbit/s. Also, the low divergence of the optical beam in a point-to-point implementation makes the transmission link more secure against undetected eavesdropping^18,19^. Furthermore, the maturity of photonic technology for terrestrial applications reduces not only developing costs and time but also the size, weight, and power consumption (SWaP) of future systems^19–21^. However, free-space optical (FSO) communication is still a topic of intense research^22–31^. Yet, so far there have been only a few satellite-ground demonstrations with data transmission: For instance, in 2008, data rates of up to 5.6 Gbit/s have been demonstrated over 1000 km in a bidirectional LEO-ground link^2,32^. Even higher distances of up to 400,000 km have been accomplished in a Moon-to-Earth transmission with 622 Mbps^21^.

Recently, several terrestrial experiments (see Supplementary Fig. S1 in Supplementary Material) demonstrated the capability of FSO communications to accomplish high data rates over small to medium distances. Line rates at and above 100 Gbit/s/λ^33–35^ over distances of up to 10.45 km^36^ have been reported. Even higher capacities of up to 1 Tbit/s/λ were achieved over distances of up to 3 m^37^ in an intra-datacentre link. To achieve such high data rates, most of these successful demonstrations relied on advanced high-order modulation formats^36–41^ and thus, required high signal-to-noise ratios, which were given by the relatively short distances. However, for future satellite links, we aim to overcome longer distances and transmit single-carrier data rates in the order of 500 Gbit/s or higher.

This raises the question as of which modulation format can address the challenges of longer distances while delivering the highest capacities. Figure 1b visualizes the trade-offs between spectral efficiency and signal-to-noise ratio (SNR) in an additive white gaussian noise channel for various QAM, ASK and PPM modulation formats (a detailed discussion is given in the Supplementary Material). The black solid curve corresponds to the Shannon’s channel capacity limit as the upper boundary. Transitioning to modulation formats with a higher spectral efficiency, will increase the capacity. However, it does require higher SNR. In free space (FS), high SNR can be realized by transmitting signals at high power, which, unlike in terrestrial fiber systems, does not suffer from the nonlinear Shannon limit^42^. However, in earth-satellite links, atmospheric conditions such as turbulence, clouds, and humidity, as well as long distances, can lead to significant FS losses (FSL) that vary over time and result in low received optical powers (ROP). Consequently, FS communication links need to be robust by being able to transmit both highest capacities under ideal conditions and under adverse low SNR situations^43^.

Moving to the left in Fig. 1b shows modulation formats with lower SNR requirements, albeit with lower spectral efficiency. The question then is as of which modulation format will transmit the highest data rates under the lowest SNR conditions. So far, the polarization-switched (PS) 4QAM (PS-QPSK) modulation format is considered the most power-efficient modulation format for coherent modulation systems^44^. It offers a 0.97 dB advantage in comparison to polarization-multiplexed (PM) 2QAM at a bit-error ratio (BER) of 1 ∙ 10^−3^. Even higher sensitivities can be accomplished with M-ary pulse-position modulation (PPM) formats. However, this comes at a very low spectral efficiency and therefore at a low link capacity^21^ and is mostly used in deep-space communication^21,45–48^. Recently, utilizing a QPSK modulation format in combination with forward-error-correction (FEC) coding schemes and a phase-sensitive amplifier (PSA) achieved a 1 photon-per-bit receiver sensitivity at data rates of 10.52 Gbit/s in a 1 m transmission experiment^49,50^. While this demonstration showed unprecedent sensitivity, the implementation of the PSA and a 100% FEC overhead adds complexity to the system whose implementation might be costly and challenging for future commercial satellite links, in particular for space terminals. Therefore, the quest for the modulation format offering the highest capacity under adverse conditions with the least complexity continues. Recently, our group introduced a new four-dimensional 2QAM (4D-BPSK) modulation format^51^ utilizing constellation modulation, which promises high throughput under the lowest SNR conditions. However, it has never been put to test in a communication link.

An additional approach to improve the overall throughput in satellite-earth links is to reduce the effects of the atmospheric impairments, i.e. FSL and atmospheric turbulences. While FSL can be partially compensated by high-power optical amplifiers and large optical telescopes, technologies such as adaptive optics (AO)^52–54^, multi-aperture^55–57^, and error correction coding^33–35^ have been investigated to mitigate the time-varying intensity fluctuations introduced by the atmospheric channel. Even though AO has shown overall higher average coupling efficiencies^15^, its effect on the signal quality in an FSO communication system with coherent modulation formats, where information is encoded onto both the amplitude and the phase of the light, is not yet clear.

In this work, we demonstrate a 53.42 km FS transmission link with single-carrier Tbit/s line-rates between the Jungfraujoch mountain top in the Swiss Alps and the Zimmerwald observatory, close to the city of Bern. This was accomplished by combining a dual-polarized 126 GBd-16QAM signal with AO at the receiver (Rx) side. In addition, we assess various coherent modulation formats and examine their suitability to achieve >100 Gbit/s/λ in ground-satellite optical links. Also, we present the novel modulation format 4D-BPSK as a solution for the reception under adverse conditions. It offers operation at record low sensitivity of 4.3 photons per bit (PPB) at a BER of 1 ∙ 10^−3^ while still capable of transmitting with >200 Gbit/s line-rates (limited by equipment). We show that, 4D-BPSK has a sensitivity advantage of 1.7 dB and 0.7 dB over PM 4QAM and PS-QPSK respectively at a BER of 1 ∙ 10^-3^. Lastly, we investigate the potential of AO to mitigate the adverse effects of the atmospheric turbulence and its impact on the signal quality of advanced modulation formats in optical satellite communication systems.

### Experimental configuration

**Fig. 2:**
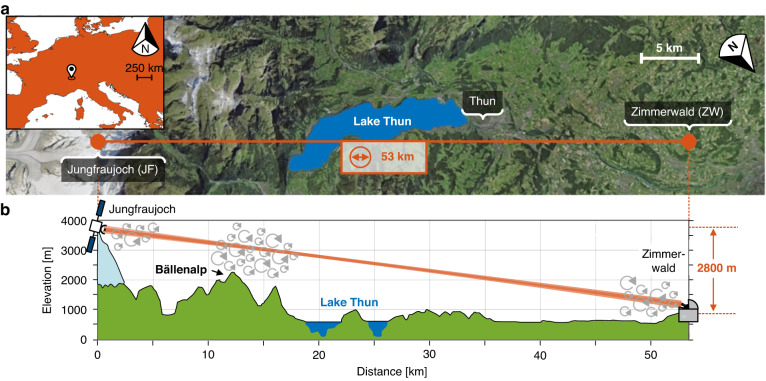
Topographical situation of the 53 km turbulent free-space optical (FSO) link. **a** Aerial view of the FSO transmission experiment with **b** the corresponding elevation profile. (swiss map data: edited from Federal Office for Topography swisstopo. Europe map data: edited from Natural Earth)

The FSO transmission experiment with an FS span of 53.42 km is shown in Fig. 2. The optical transmitter is located at the Jungfrau East Ridge building (3700 m above sea-level) at the glacier Jungfrau, while the optical Rx is situated in the Zimmerwald Observatory of University of Bern (895 m above sea-level). The optical beam propagates through the atmosphere with a total elevation of 2800 m. Since the beam stays inside the atmosphere close to the surface and has to pass turbulent mountainous areas and a lake with an all-different thermal situation (see Fig. 2), the test conditions represent a worst-case scenario for a ground-GEO link - despite the total smaller distance.

**Fig. 3:**
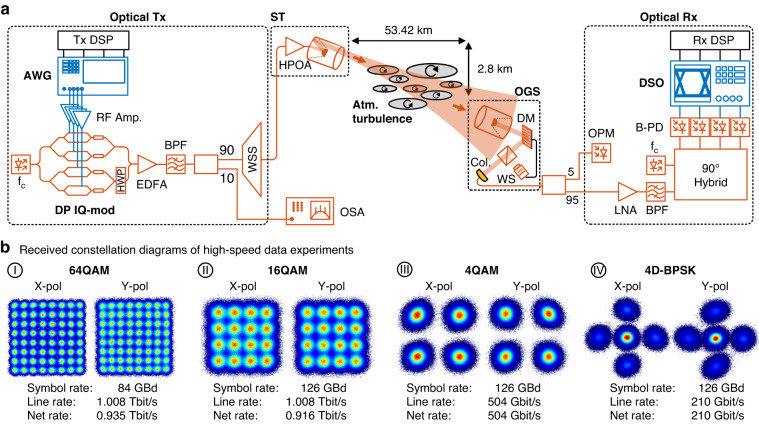
A 53 km free-space optical (FSO) link with high-speed data transmission. **a** Experimental setup; First, a dual-polarized complex-modulated signal is generated by an arbitrary waveform generator (AWG) and mixed onto an optical carrier fc (1550 nm) by means of a DP IQ-modulator. Afterwards it is optically equalized by a wavelength selective switch (WSS) and fed to the space terminal (ST). Subsequently, the signal is transmitted over a FSO distance of 53 km through a turbulent atmospheric channel. At the receiver side, the phase-distorted wavefront is corrected in an adaptive optics system consisting of a wavefront sensor (WS) and a deformable mirror (DM), and coupled to a single mode fiber. After pre-amplification in low-noise amplifiers (LNA), the data signal is mapped back to baseband in the optical coherent receiver consisting of a local oscillator, a 90°-Hybrid and balanced photodetectors (B-PD). Finally, the digital signal is assessed by a standard offline digital signal processing stage. **b** Received constellation diagrams of high-speed data transmission are shown, achieving single-carrier symbol-rates of up to 126 GBaud. This results line rates of up to 1.008 Tbit/s by means of advanced modulation formats. (Amp amplifier, Atm atmosphere, AWG arbitrary waveform generator, B-PD balanced photodetector, BPF band pass filter, Col collimator, DM deformable mirror, DSP digital signal processing, DP dual polarization, DSO digital sampling oscilloscope, IQ-mod in-phase quadrature modulator, EDFA erbium-doped fiber amplifier, HPOA high-power optical amplifier, HWP half wave plate, LNA low noise amplifier, OGS optical ground station, OPM optical power meter, Rx receiver, ST space terminal, WS wave-front sensor, WSS wavelength-selective switch.)

The experimental setup of the FSO communication link is illustrated in Fig. 3a. Key elements of the link are advanced optical modulation formats, the AO system and the low-noise-amplifier (LNA) optical coherent Rx front-end. In the optical transmitter (Tx) at Jungfraujoch, the electrical signal, consisting of a QAM signal with a random-bit sequence of >54,400 bits, is encoded onto a 1550 nm optical carrier by means of a dual polarization (DP) in-phase and quadrature-phase (IQ) modulator. After optical equalization, the signal enters the space terminal, where it is boosted by a high-power optical amplifier to 27 dBm to compensate for the FSL and divergence of the optical beam. The optical beam then propagates through the 53 km long turbulent FS channel with a total elevation of 2800 m. After reception at the optical ground station in Zimmerwald, the phase-distorted wavefront of the optical beam is corrected by the AO system, consisting of a Shack–Hartmann wavefront sensor and a deformable mirror (DM) and a real-time computer (RTC). The frequency of the AO control loop is 1.5 kHz. This is needed, as the coherence time of the 53 km channel was measured to be in the order of a few milliseconds. The signal is then coupled back to a single-mode fiber. Subsequently, the optical signal is amplified by low-noise erbium-doped fiber amplifiers (noise figures of the EDFAs are 3.4 dB and 3.7 dB respectively), band-pass-filtered and down-converted to baseband by a local oscillator in the optical coherent Rx. Finally, the sampled signal is evaluated by an offline linear standard digital signal processing (DSP) stage (see Methods section). Figure 3b shows the received constellation diagrams of advanced modulation formats at highest speeds. Single-carrier line-rates of up to 1.008 Tbit/s have been transmitted. The results of the FSO link are now described in more detail.

## Results

**Fig. 4:**
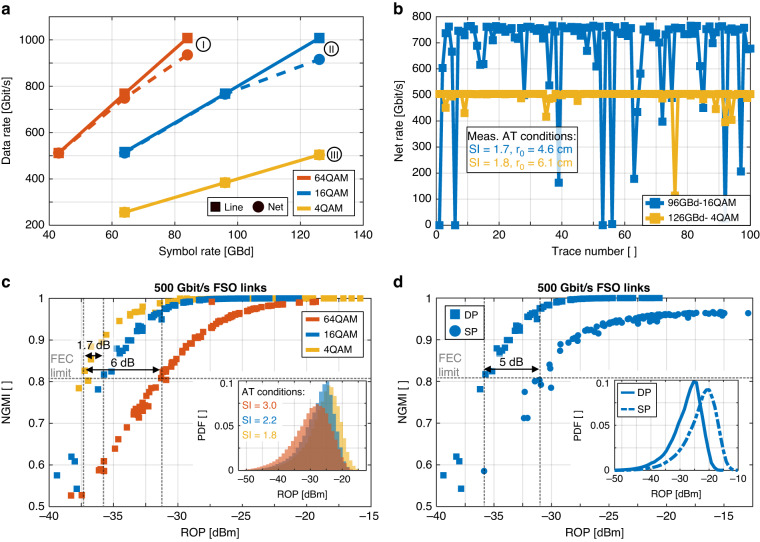
Results of the 53 km FSO Tbit/s single carrier data transmission experiments. **a** Line rates (square symbols) of up to 1Tbit/s and net rates (circle symbols) as a function of symbol rate for different polarization-multiplexed (PM) QAM modulation formats. Highest data rates have been obtained with PM 126GBd-16QAM and PM 84GBd-64QAM modulation formats. One may also notice that transmission of 1 Tbit/s with 16QAM and 4QAM is a challenge as the transmitter lacks sufficient bandwidth. **b** Net rates as a function of 100 consecutive measurements (every 1.5 s) for PM 96GBd-16QAM and PM 124GBd-4QAM under strong-atmospheric-turbulence conditions with scintillation values SI of 1.75 and fried parameters r0 of 5.4 cm. The power fluctuations of the turbulent channel leads to a higher risk of losing data at higher modulation formats. One would rather resort to lower modulation formats such as 4QAM at highest symbol rates. **c** Normalized GMI (NGMI) as a function for received optical power (ROP), coupled to the fiber, for different PM QAM modulation formats at fixed line-rates of 500 Gbit/s. The dashed line indicates the threshold for a 0.75 FEC code rate. The inset shows the corresponding ROP probability distribution for each modulation format. Despite bandwidth constraints, 4QAM has still a sensitivity advantage of up to 1.7 dB and 6 dB over 16QAM and 64QAM and enables 500 Gbit/s transmission above the 0.75 FEC rate at ROP as low as −37 dBm. **d** NGMI as function of ROP for a single-polarization (SP) and a dual-polarization (DP) 16QAM modulation format at fixed line rates of 500 Gbit/s. The inset shows the corresponding ROP probability distribution for both measurements. Polarization-division multiplexing (PDM) is a technique that can double the spectral efficiency, allowing the same data rate to be transmitted using half the bandwidth. Theoretically, sending the information in a SP results in the same NGMI over ROP behavior. However, in practice, bandwidth limitations can limit the performance of SP transmission. For this reason, using PDM is attractive and results in a power gain of up to 5 dB

Figure 4a plots the line rate and highest achievable net-rates as function of symbol rate for different PM modulation formats. The line rates are given by multiplying the symbol rates with the number of polarizations and log_2_(*M*), where *M* is the number of symbols in the constellation. The net rates can be derived by multiplying the symbol rates with the number of polarizations and the experimentally found generalized mutual information (GMI). It can be seen that the highest line rates of up to 1.008 Tbit/s have been obtained by choosing higher-order modulation formats such as 64QAM and 16QAM, resulting into net rates of 935 and 910 Gbit/s respectively. Such high transmissions are only possible because in a FSO channel one can transmit high powers without being penalized by the nonlinear Shannon limit^42^ - unlike in a fiber link. The high powers are needed to guarantee a sufficiently high SNR as necessary for transmission of advanced modulation formats, see Fig. 1b. Conversely, sending lower-order modulation formats at higher symbol rates is penalized by the bandwidth limitations of the equipment, here the 38 GHz bandwidth of the IQ-modulator, resulting in a lower information rate and thus, net rate for 16QAM and 4QAM in comparison to 64QAM. The constellation diagrams belonging to the plots I, II and III are shown in Fig. 3b.

However, transmitting higher modulation formats comes at the price of a higher risk of losing data under turbulent conditions. This effect of the atmosphere can be seen in the calculated net rates for 100 consecutive data measurements (every 1.5 s) for PM 96GBd-16QAM and PM 126GBd-4QAM in Fig. 4b. The AO can only mitigate atmospheric turbulences to some extent (see below) and the residual power fluctuations in the Rx lead to time-varying SNRs and net rates. For ROP below the FEC threshold, the measured data sequence cannot be evaluated and is completely lost. Both measurements were conducted under high-perturbation conditions. In the outdoor demonstration, the scintillation index (*SI*) and the Fried’s coherence length *r*_0_^58^ are used as figure of merits to evaluate the present atmospheric turbulence conditions. The *SI*, defined as the ratio of the standard deviation of the intensity *σ*_*I*_ to the average intensity of the beam <*I*>, was calculated based on the beam’s intensity *I*(*x,y, t*) in the pupil plane measured by Shack–Hartmann wavefront sensor. During the outdoor demonstration, the measured *SI* showed temporal variability and ranged from 1 to 4 over a few hours (see Supplementary plot S5). In contrast, typical scintillation indices *SI* in GEO-earth links for worst-case scenarios are <1^59^. Here, during the outdoor demo, data measurements with *SI* of up to 4 have been performed successfully. The Fried’s coherence length *r*_0_ characterizes the phase spatial correlation^58^. Small r_0_ means strong turbulence induced phase variations. It is estimated through post-processing of AO data (Shack–Hartmann data and deformable mirror commands)^59,60^. Values of *r*_0_ ~ 4 cm are in the same order of magnitude as expected in a real earth-GEO transmission scenario^59^.

Turbulences affect the ROP and thereby impact the transmitted data. The plot in Fig. 4c shows the normalized GMI (NGMI) as a function of the ROP for the 3 modulation formats at or adjusted to 500 Gbit/s. Despite a higher symbol-rate and operation at the bandwidth limits of the 4QAM modulation format, 4QAM still has a sensitivity advantage of up to 1.7 dB and 6 dB over 16QAM and 64QAM for NGMI values at and above 0.81. Even higher sensitivity advantages of 3.7 and 6.9 dB, see Supplementary plot Fig. S2a, may be achievable in the absence of bandwidth constraints. The NGMI of 0.81 constitutes a threshold for an ideal code rate at which data can be transmitted without an error if a proper FEC code would be applied. Practical code-rates are somewhat lower. E.g. in ref. ^61^ a concatenated tail-biting soft decision (SD) code and a hard decision (HD) code with a code rate of 0.75 is reported that provides error-free transmission for signals with an NGMI of 0.81. For fixed line rates of 500 Gbit/s, this results into practically achievable net rates of 375 Gbit/s. The inset in Fig. 4c shows the probability density function of the ROP during transmission for each of the 3 modulation formats (64QAM, 16QAM, and 4QAM). One can see that turbulences lead to power fluctuations by more than 20 dB during a measurement series despite the AO correction. As the ROP fluctuates, errors occur and degrade the achievable net rate.

Polarization division multiplexing has given us another degree of freedom to transmit higher data rates. It doubles the spectral efficiency [bit/s/Hz], meaning that twice the information can be transmitted for a given system bandwidth. In theory, sending the information in a single polarization (SP) or sending it as a DP with half the symbol rate and half the power in each polarization should lead to the same NGMI versus ROP plot. However, in experiments, see Fig. 4d, we find a 5 dB penalty for sending the same 500 Gbit/s once as an SP 124 GBd over sending it as a DP 64 GBd-16QAM. The penalty is due to the operation at twice of the speed and the related hardware limitations. Consequently, resorting to PM modulation is advantageous as it enables operation at lower bandwidth. As an interesting detail, it has been found that the FS channel’s polarization state does not change with time, see Supplementary plot Fig. S4a. I.e. deploying a DP over a SP does not really require a fast PDM signal processing algorithm as in a fiber channel.

In summary, the use of high-order PM modulation formats enables Tbit/s transmission. However, this requires a high SNR and is a good solution if transmission at high powers is possible and channel losses are small. Unfortunately, low SNR conditions are frequent, and the question then is which modulation format can transmit the highest data rates under lowest SNR conditions.

To determine the best modulation format for reliable communication at the highest capacity with very low power, we next assess the sensitivity of various advanced modulation formats in the 53 km FSO link. In this study, we not only evaluate PM-QAM modulation formats, but also consider two additional 4D modulation formats: PS-QPSK and 4D-BPSK. The latter extends the traditional BPSK (2QAM) by constellation modulation. In constellation modulation we first encode information by selecting any of *C* possible constellations. Here, we select any of 4 possible BPSK constellations in the 4-dimensional space (a BPSK constellation can be arranged vertically or horizontally in the complex plane and can be in either of two polarizations). See Supplementary Fig. S3a. Selecting any of *C* = 4 constellations allows one to encode log_2_ *C* = 2 bits. Secondly, within the constellation one now can encode information by selecting any of *M* symbols – corresponding to log_2_ *M* bits. For BPSK with *M* = 2 for this adds log_2_ *M* = 1 bit per symbol. Lastly, we maintain the same constellation for *L* = 3 times – while the modulation encoding of symbols continues. This increases the SNR tolerance of constellation switching but comes at a price of a lowering the spectral efficiency of constellation modulation to log_2_ *C/L*. Ultimately, the overall spectral efficiency of constellation modulation provides a spectral efficiency per polarization *P* of^51^

$${SE}\,=\left[\frac{{\rm{lo}}{{\rm{g}}}_{2}C\,}{L}+{{{\log }}}_{2}M\right]\cdot \frac{1}{P}$$

For a BPSK modulation format with *M* = 2, and *L* = 3 and a selection of *C* = 4 constellations we receive a SE of $0.8\bar{3}$ bit/s/Hz/Pol. (A detailed description of the 4D-BPSK encoding with visualization is given in the Supplementary material). With this choice, simulations predict SNR-per-bit advantages of 1.7 dB and 0.75 dB over 4QAM and PS-QPSK at a BER of 1 ∙ 10^−3^ (see Supplementary plot Fig. S3). This would make 4D-BPSK one of the most SNR-tolerant modulation format with a SE close to 1.

**Fig. 5:**
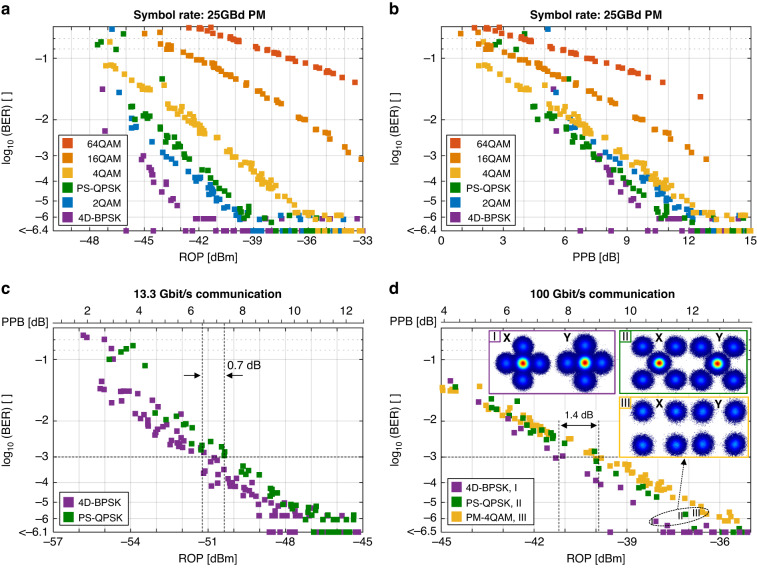
Sensitivity of advanced modulation formats in the 53 km FSO link. Bit-error ratio (BER) as a function of **a** ROP and **b** photon-per-bit (PPB) for polarization-multiplexed QAM modulation format at symbol-rates of 25 GBd. The 4D modulation formats PS-QSPK and 4D-BPSK are also investigated. We find that they need the lowest number of PPB for a given BER. This is in agreement with the theory (see Supplementary material). BER versus ROP at fixed line rates in the order of **c** 13.3 Gbit/s/λ and **d** 100 Gbit/s/λsee Methods section). 4D-BPSK shows to be the most power-efficient modulation format, with measured sensitivity advantage of 0.7 dB and 1.4 dB over PS-QPSK and PM 4QAM respectively at a BER of 1 ∙ 10^−3^. In combination with a low-noise amplifier optical coherent Rx front-end, it is capable to transmit at ROP as low as −51.3 dBm and −41.2 dBm respectively, only 1.4 dB from the ideal shot noise limit

Figure 5a shows the BER as a function of ROP for various modulation formats at symbol-rates of 25 GBd, measured after the 53 km turbulent FS channel. It can be seen that the 4D-BPSK modulation format permits to receive 25 GBd of information with as little as −45 dBm at a BER of 1 ∙ 10^−3^ after 53 km FSO transmission. Now, since the signals in Fig. 5a have different SE, a fairer comparison would be plotting the results of the same experiment with respect to photons per bit (PPB). Figure 5b shows the BER as a function of PPB, disclosing the sensitivity differences between the modulation formats. It can be seen that 4D-BPSK and PS-QPSK offer the highest sensitivity. The many purple square symbols at the bottom show 4D-BPSK experiments for which within a set of ~800,000 symbols no error was found. Based on the large number of purple symbols at the bottom, it appears that 4D-BPSK is indeed delivering higher signal quality for less power. In practice, a provider will have to transmit a variable data rate between 10 and 100 Gbit/s/λ. The question thus would be as of which modulation would provide error-free transmission under the lowest SNR conditions. The results for fixed line rates of 13.3 and 100 Gbit/s are given in Fig. 5c, d, respectively. Evaluations of these experiments show that 4D-BPSK offers an advantage of 1.4 dB over QPSK and 0.7 dB over PS-QPSK at a BER of 1 ∙ 10^−3^. In addition, the experiments in c and d show reliable 13.3 Gbit/s data communication at ROP down to −51.3 dBm and reliable 100 Gbit/s data communication with −41.2 dBm, respectively, achieving a high sensitivity of only 6.4 dB PPB, see Fig. 5c. All the results indicate that 4D-BSPK is indeed one of the most noise-tolerant modulation formats.

**Fig. 6:**
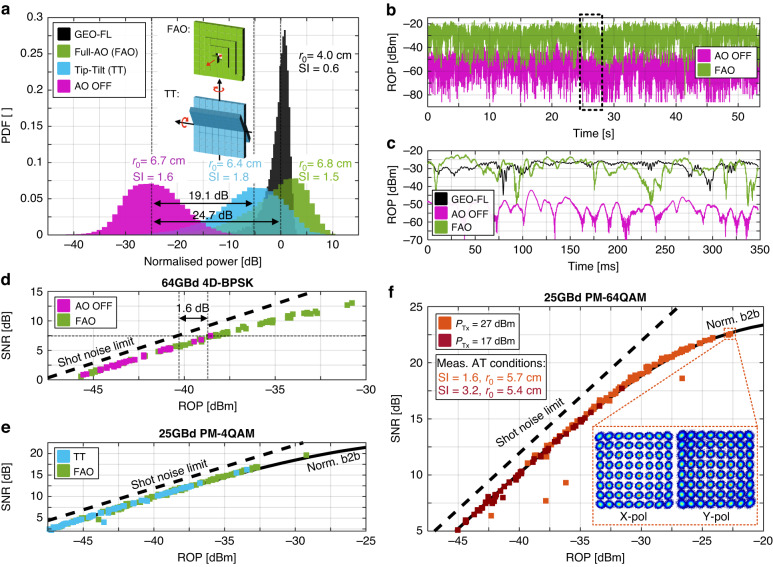
Investigation into the performance of adaptive optics (AO). **a** Probability density function (PDF) as a function of normalized power for different adaptive optics settings, namely full adaptive optics correction (FAO, green), tip-tilt correction (TT. blue) and no correction (OFF, pink). For comparison, we plot the expected PDF for a worst case scenario of a GEO feeder-uplink (FL) with FAO (black). We find that the operation in FAO setting yields to 5.6 dB gain and 24.7 dB gain in median received optical power (ROP) over TT setting and OFF setting, respectively. Comparison with the reference GEO-FL shows that experiments have been performed under worst case turbulent conditions. The measurement in FAO configuration with measured *SI* = 1.5 is way more turbulent than the reference GEO-FL. **b** ROP versus time for the FAO and OFF settings on second scale. **c** The zoom-in of (**b**) illustrates the coherent time of the channel to be in the order of a few milliseconds. Here again a representative time evolution of a worst-case GEO-FL scenario is plotted in black and adjusted to the median of the FAO ROP. Employing FAO increases the median coupled power into the Rx and reduces the frequency of power fading’s. **d,** **e** The effect of adaptive optics is to provide a ROP advantage. Other than that there is no penalty for turning on AO. This can be seen by the fact that the ROP versus SNR measurements with and without AO plots overlap without any discontinuity. The same applies for tip tilt (TT) AO. The dashed curve corresponds to the ideal shot-noise limit and the solid line shows the results of back-to-back (b2b) measurements in the lab, normalized to 25 GBd. The FSO measurements are also in agreement with the b2b results, revealing that AO nor the turbulent channel lead to an additional SNR penalty. **f** SNR as a function of ROP for different Tx launch powers *P*_Tx_ using the high-order modulation format 64QAM. We find that one can send higher powers to achieve higher SNR values as there exists no upper nonlinear limit in the FSO channel

In the next step, we investigate the benefit of adaptive optics (AO) on the performance of the FSO communication links. Our AO system gave access to three different operation settings, see inset Fig. 6a: Full AO compensation (FAO), which steers the 97-actuators of the DM individually; tip-tilt correction (TT), where the configuration of the 97 actuators is restricted to mimic the correction of a fast tip-tilt mirror (adaptions around 2 axes); and OFF, which disables the active correction and only static aberrations are corrected for. FAO correction employs all 97 actuators to correct 56 phase modes. In general, the larger the ratio between AO telescope diameter *D* and the Fried coherence length *r*_0_, the more phase modes need to be taken into account for an efficient correction^15^. Consequently, for a *D* of 35 cm and r0 in the order of ~4 cm, we expect to see a better performance for FAO correction than just TT. Figure 6a shows the probability density function for the three different adaptive optics settings as a function of power, normalized to the median of ROP in FAO configuration. All the tests were performed under similar turbulent atmospheric conditions, see Fried’s coherence length *r*_0_ and scintillation index *SI* values in the insets. In addition, we also show the expected PDF of a worst-case GEO feeder-uplink (FL), see Methods section. Here ROP variations stem from residual phase variations after FAO and scintillation. The distributions clearly illustrate that the median power distribution increases by 19.1 dB when TT is applied, while it improves up to 24.7 dB, when FAO is used. To elaborate further, in Fig. 6b, c, the ROP of these measurements are plotted over time on two different time scales. Figure 6b shows the ROP on a second time scale, while Fig. 6c shows the ROP on a millisecond time scale. It can be seen that FAO is capable of reducing the frequency and depths of power fadings^62^ with respect to OFF, see Fig. 6c. Figure 6a–c demonstrates that using AO results in a power advantage. Furthermore, we find that the channel, here with only *SI* of 1.7, is much more turbulent than expected in the worst-case scenario of a GEO-FL. In the next step, we determine if the use of AO introduces a modulation effect, or penalty, on advanced modulation formats. Advanced modulation formats are sensitive to phase distortions and the challenge in an AO correction is to correctly perform the coherent combinations of the amplitudes and phases of the incoming wavefront. Therefore, we investigate the potential performance impacts on the SNR for different AO settings as a function of ROP in Fig. 6d, e. To gain a clearer understanding of the data points and their level of agreement, we compare d the FAO setting with the OFF setting once for a 106 Gbit/s 4D-BPSK modulation format and we compare e the FAO setting with the TT setting for a 100 Gbit/s PM-4QAM signal. The dashed curves correspond to the ideal shot-noise limit, while the solid line shows the results of back-to-back (b2b) measurements in the lab. It can be seen that the plots, which were taken in TT-configuration and OFF-configuration, respectively, overlap when turning on the FAO correction. There is no SNR discontinuity when turning on FAO corrections. Moreover, one may observe in Fig. 6e that the FSO results and the b2b plots (black line) taken in the labs perfectly overlap. The FSO measurement matches those obtained from the b2b measurements, with deviations as low as 1.6 dB from the ideal shot noise limit. This demonstrates that the FS-span only adds attenuation and no other degradation. This is a remarkable result as the FSO link was rather turbulent. Figure 6f shows the SNR as a function of ROP for different Tx launch powers *P*_Tx_ in the FSO link using the high-order modulation format 64QAM. The solid black line shows the SNR versus ROP of the b2b experiment in the lab. Again, we find a perfect overlap of FSO with the back-to-back experiment. At higher powers, one may now notice that the b2b as well as the FSO measurements deviate from the shot noise limitation and converge to a fixed SNR value. This drop-off is due to the nonlinear limit of the transmitter and the quantization noise of the AWG and DSO. The absence of an SNR penalty in FSO for higher optical launch powers confirms that the performance in FSO links is not upper-limited by the nonlinear Shannon limit.

## Discussion

In summary, we have shown Tbit/s line rate transmission over a 53.42 km turbulent FS channel, corresponding to net rates of up to 0.94 Tbit/s. This was accomplished by employing polarization-multiplexed higher-order modulation formats at the highest symbol rates and a Full Adaptive Optical (FAO) system that enabled us to achieve high SNR values. We have shown that adaptive optical filtering can handle coherent modulation formats even under strong-turbulence conditions. Furthermore, the novel four-dimensional BPSK (4D-BPSK) modulation format based on constellation modulation was tested for its ability to handle low SNR conditions. 4D-BPSK allows to transmit high data rates at the lowest ROP, with a measured sensitivity advantage of 0.7 dB over PS-QPSK at a BER of 1 ∙ 10^−3^. More precisely, 13.3 Gbit/s and 100 Gbit/s of data were transmitted at a received optical power of −51.3 dBm and −41.2 dBm. All of these results are good news for the space communications community. Ultimately, attenuation is the only challenge that needs to be overcome in view of a large-scale free-space optical deployment and even this limit can be stretched by the introduction of a new low SNR modulation format.

## Methods

### Free-space optical communication link experiment

The baseband data signal has been generated by 128 GSa/s digital-to-analog converter (DAC) with a 3-dBbandwidth of 65 GHz and 8 bit vertical resolution. The bandwidth limitation of the 38 GHz DP IQ-modulator has been compensated by a wavelength selective switch, acting as an optical filter to flatten the power spectral density. The equalized signal has then been amplified to 27 dBm and coupled to FS by a space terminal emulator. The terminal uses a 4.2 cm sub-aperture of a 20 cm diameter telescope for its outgoing beam expansion. The divergence of the optical beam was measured to be approximately 100 μrad (half-angle). The ground station consisted of a 35 cm telescope and an adaptive optic system to correct the phase-front error of the perturbated EM wave: A 8 × 8 sub-aperture wavefront sensor analyses the incoming wave and a deformable mirror with 97 actuators has then been used to shape the wavefront back to a planar wave, which is known to be ideal for FS-to-Fiber coupling. As the coherent time of the channel is in the order of a few milliseconds, the configuration of the deformable mirrors was updated at a frequency of 1.5 kHz (in a feedback loop scheme). After amplification in the EDFAs and down-mixing by the local oscillator in the Balanced PDs, the baseband signal is captured by a 256 GSa/s analog-to-digital converter (ADC) with a 3**-**dB bandwidth of 113 GHz and a 10 bit vertical resolution.

### DSP chain

Offline DSP for waveform generation and evaluation has been applied. The transmitter DSP first generated a random bit sequence and mapped the bits to the symbols for the different constellation/modulation formats. Afterward, the digital signal is square-root-raised-cosine pulse shaped. Symbol rates above or equal to 96 GBd have been pre-distorted by a linear FIR filter to compensate for the low-pass characteristics of the electrical transmitter components, i.e. AWG, RF amplifier and RF cables. In the receiver DSP, the digitized received signal has first been de-skewed with respect to each other and orthonormalized to compensate for the imperfections of the Rx frontend. After matched filtering, the timing of the signal has been recovered by a modified Godard algorithm^63^. For polarization-demultiplexing, a 2 × 2 feed-forward equalizer has been used where the filter coefficients have been blindly trained based on a constant modulus algorithm (CMA) with a steepest gradient descent cost function. Modifications to the CMA’s cost function were necessary for PS-QPSK and 4D-BPSK, as outlined in ref. ^64^, and for BPSK, as described in ref. ^65^. For higher-order modulation formats, a radium distance equalizer (RDE) has been additionally applied. A 4^th^ power algorithm has removed the carrier frequency offset originating from the frequency drifts between the signal laser and the local oscillator laser, and a phase recovery algorithm based on a blind phase search has compensated for the laser phase noise. In the next step, a final 2 × 2 feed-forward equalizer based on data-aided least-mean square algorithm has been deployed to combat the bandwidth limitations of the electrical and optical components of the receiver front-end. Finally, the symbols have been mapped back to the binary string and eventually SNR, BER and other figures of merit have been evaluated.

### Sensitivity measurements

The exact symbol-rates in Fig. 5c are 8 GBd for 4D-BPSK and 4 GBd for PS-QPSK, which correspond to line rates of 13.33 Gbit/s and 12 Gbit/s, respectively. As a result, PS-QPSK incurs a ROP penalty of 10∙log_10_(13.$\bar{33}$/12) = 0.45 dB due to its higher bit rate. To make the plots comparable, we have offset the plot in Fig. 5c that represents PS-QPSK to compensate for the penalty it incurs due to its higher bit rate.

Similarly, the exact symbol-rates in Fig. 5d correspond to 60 GBd for 4D-BPSK, 35.5 GBd for PS-QPSK and 25 GBd for PM-QPSK, resulting in net-rates of 99.$\bar{99}$ Gbit/s, 106.5 Gbit/s and 100 Gbit/s respectively. In order to compare the three experiments at 100 Gbit/s, the PS-QPSK plot has been offset by 10∙log_10_(106.5/99.$\bar{99}$) = 0.27 dB to the right in order to fairly compare the three formats at same data rates.

### GEO feeder link

The simulations consider a GEO feeder link with an elevation angle of 30 degrees. In the uplink scenario, an isoplanatic angle of 6.8 μrad and a point ahead angle of 18.5 μrad is assumed over the total distance of ~38,000 km. The performance of the AO system is based on the measurements with the existing FEEDELIO optical ground station^59^. The bottom wind profile are estimated to be 10 m/s on ground and 30 m/s at high altitude. For the vertical turbulence, we created a hybrid database of atmospheric turbulence by combining data from instrumental measurements in Paranal (Chile)^66^and Cn² measurements in Tenerife^67^. The Monin Obukhov Similitude (MOS) theory finally makes the connection between the ground layer and free atmosphere. We find that the scintillation index *SI* in GEO Feeder cases to almost never exceed 1 (probability <1%).

## Supplementary information

Supplementary Information

## Data Availability

The data that support the plots within this paper and other findings of this study are available from the corresponding author upon reasonable request. The landscape maps and elevation profile in Fig. 2 came from the publicly available datasets swisstopo and Natural Earth. Permission for re-use was received from the data providers for use of datasets in PNG.
