# Supplementary material for: Tbit/s line-rate satellite feeder links enabled by coherent modulation and full-adaptive optics
Source: Light Sci Appl. 2023 Jun 20;12:153. doi: 10.1038/s41377-023-01201-7 (PMC10282091; doi:10.1038/s41377-023-01201-7)
Supplement: Supplementary file 1 — Supplementary Information [file 41377_2023_1201_MOESM1_ESM.pdf]

Supplementary Information for

Tbit/s Line-Rate Satellite Feeder Links Enabled by Coherent Modulation and Full-Adaptive Optics

Yannik Horst[1], Bertold Ian Bitachon[1], Laurenz Kulmer[1], Jannik Brun[1], Tobias Blatter[1], Jean-Marc Conan[2], Aurélie Montmerle-Bonnefois[2], Joseph Montri[2], Béatrice Sorrente[2], Caroline B. Lim[2,*], Nicolas Védrenne[2], Daniel Matter[3], Loann Pommarel[3], Benedikt Baeuerle[1,4], Juerg Leuthold[1,4]

[1] ETH Zurich, Institute of Electromagnetic Fields (IEF), Gloriastrasse 35, 8092 Zürich, Switzerland
[2] ONERA, DOTA, Paris Saclay University, F-92322 Châtillon, France
[3] Thales Alenia Space in Switzerland, Schaffhauserstrasse 580, Zürich 8052, Switzerland
[4] Polariton Technologies AG, 8803 Rüschlikon, Switzerland
** Currently with LNE-SYRTE, Observatoire de Paris, Paris, France*

State of the Art

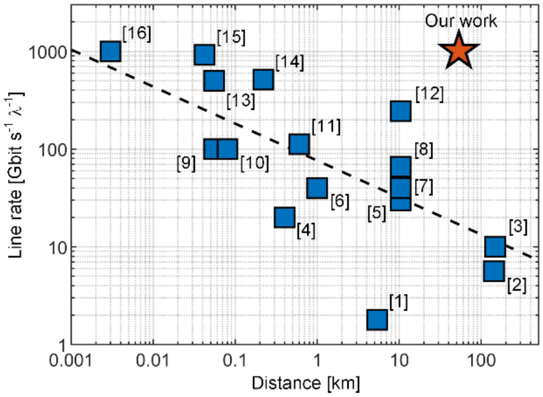

Fig. S1. Selection of reported terrestrial free-space optical communication links and current highest performance using coherent and direct modulation/detection schemes [1-16].

In the last decade, multiple terrestrial free-space optical (FSO) transmission links [1-16] demonstrated single-carrier line-rates at and above 100 Gbit s^{-1} [8-12] while overcoming short to medium distances, see Fig. S1. For instances, researchers of the DLR in Germany performed a 10.45 km demo under worst-case turbulence condition and achieved up to 240 Gbit s^{-1} λ^{-1} line-rate [12]. Recently, Fernandes and colleagues accomplished a 1 Tbit s^{-1} link over 3 m for future datacentre interconnects [16]. Mitigation techniques such as adaptive optics made it possible to overcome free-space distances of 147 km at line-rates of up to 10 Gbit s^{-1} [3]. These successful demonstrations show that FSO links can achieve high spectral efficiencies (up to 6 bit s^{-1} Hz^{-1}) without being restricted by a nonlinear limit. However, data-rates decrease at longer distances (as shown by the dashed line). Consequently, to accurately mimic a ground-satellite link, it is necessary to improve FSO links to allow for high capacity single-channel transmission over long, oblique distances.

Advanced Modulation Formats for FSO communication

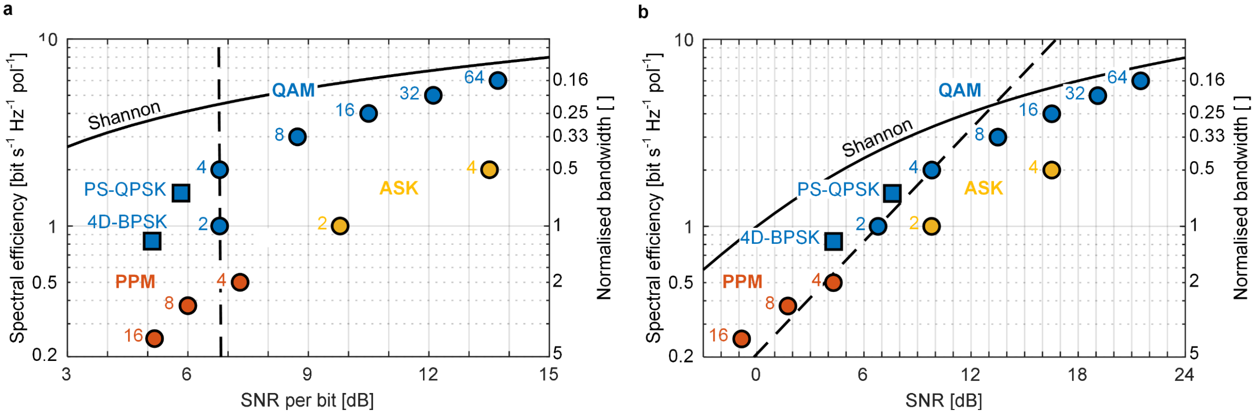

Fig. S2 Sensitivity investigation of advanced optical modulation formats. Spectral efficiency versus **a** SNR/bit and **b** SNR for QAM, ASK and PPM modulation. In addition, two 4D modulation formats, namely PS-QPSK and 4D-BPSK are plotted. The solid curve corresponds to the Shannon limit while the dashed curve is a reference line.

Fig. S2 visualizes the trade-offs between spectral efficiency and sensitivity in an additive white gaussian noise (AWGN) channel for PAM, PPM and QAM modulation formats. The black solid curve corresponds to the Shannon channel capacity limit as the upper boundary, while the dashed line represents an auxiliary line for the baseline modulation format BPSK (2QAM) selected here. Here, an idealized forward error correction (FEC) coding is assumed that provide full channel capacity at a bit-error-rate (BER) of $1\cdot10^{-3}$. The plot Fig. S2a needs to be understood as follows: Moving from one modulation format to the next along the dashed line will not change the SNR/bit ratio. I.e. when switching from a 2QAM to a 4QAM signal the bits/symbol will increase by a factor 2, yet at the same time the SNR requirement will also increase by a factor 2, which results in an unchanged SNR/bit ratio. In other words, switching from 2QAM to a 4QAM will increase the spectral efficiency by a factor two, yet as the SNR requirement increases by a factor 2 as well one would lower the symbol-rate by a factor 2 maintain the SNR/bit. Therefore, there

is no net-advantage in moving along the vertical axis – except that smaller symbol rates may be chosen to better the spectral efficiency. In a bandwidth-limited system one would resort to advanced modulation formats.

However, moving to the right in this plot comes at the price of higher SNR requirements. This is worth the price if higher spectral efficiency and/or smaller bandwidth is needed. For instance, switching from a 4QAM to a 16QAM modulation format doubles the spectral efficiency, yet it requires an SNR/bit improvement of ~3.7 dB. The higher SNR requirements can be met by transmitting the data with a 3.7 dB higher power laser. However, if constraints apply to the maximum power, then a modulation format to the left should be selected. Moving to the left gives relaxed SNR requirements. It might come at the price of lower spectral efficiency though. The plot shows that transitioning to e.g. the polarization-switched PS-QPSK modulation format can be transmitted with relaxed SNR. PS-QPSK modulation so far is considered the most power-efficient modulation format for uncoded, coherent modulation systems [17]. It offers a 0.97 dB advantage in comparison to polarization-multiplexed 2QAM at a bit-error-ratio of $1\cdot10^{-3}$, and is selected, when SNR is low. The plot also shows the SNR/bit of the newly introduced 4D-BPSK constellation modulation format. This modulation format is shown to even offer a 1.7 dB SNR/bit improvement over 2QAM and 0.75 dB over PS-QPSK. In fact, from the plot Fig. S2a it can be seen that 4D-BPSK is very far to the left, which makes it one of the most sensitive modulation format.

Even higher sensitivities can be accomplished with M-ary pulse position modulation (PPM) formats. However, this comes at an overly large expense of capacity of the link. For example, with 16-PPM the spectral efficiency drops to 0.25 bit/s/Hz while the SNR/bit remains unchanged. Transitioning to 64-PPM, one would take a -10 dB penalty in spectral efficiency to obtain a ~3 dB sensitivity advantage in comparison to 2QAM. Although available symbol-rates doubled in the last 5 years [18], or even 10-fold in the last ten years [19], by using PPM-modulation formats it will be challenging to reach data-rates of 100 Gbit/s and higher in the near future.

The plot also shows the SNR/bit requirements for 2ASK and 4ASK signals, which are the focus of recent space programs such as OSIRIS [20] , Optel-μ [21] and SOTA [22]. These modulation formats are attractive as they come with a simple receiver architecture. Yet it can be seen that the SNR/bit requirements are also considerably higher for the two PAM modulation formats if compared against e.g. a 2 or 4QAM, for equal spectral efficiencies.

The plot Fig. S2b shows the spectral efficiency plotted against the SNR. When transitioning from one modulation format to another, they exhibit equivalent power-efficiency if a doubling of the spectral efficiency is penalized by a factor 2 in SNR requirement. All modulation formats lying on the dashed auxiliary line have the same power-efficiency as our baseline modulation format 2QAM. If a modulation format lies to left of the auxiliary line, it is deemed to be more power-efficient than 2QAM.

Constellation Modulation: 4D-BPSK

In the next step, we present the implementation of 4D-BPSK. In 4D-BPSK, we introduce constellation modulation as an additional degree of freedom to encode information. For instance, BPSK can be encoded in the complex plane by arranging the symbols on the real or imaginary axis. So, there are two possible BPSK constellations in the x-polarization. Moreover, there are two more BPSK constellation in the y-polarization. Thus, in total we can pick between any of 4 possible BPSK constellations the complex polarization space, see Fig. S3a. The expansion to the 4-dimensional space results in the ability to encode two additional bits, as shown by the plot Fig. S3a with its four different constellations. Fig. S3b presents a schematic representation of the constellation modulation encoder for 4D-BPSK. It can be understood as follows: The input bit stream (e.g. 010111) is segmented into two blocks: E.g. the first 2 bits (i.e. the 01 sequence) is encoded by selecting one out of the of C=4 BPSK constellations (here constellation c_2 for 01). The next 3 bits (e.g. 011) are encoded as BPSK signals within the chosen constellation. By now, we have mapped 5 bits onto 3 symbols. The absolute distance from the M =2 symbols in complex space is 2 (with respect to an amplitude normalized to 1). However, the absolute distance between two constellations is only $\sqrt{2}$. We therefore maintain the constellation for L=3 times to increase the SNR of the constellation encoded information. Finally, the total information encoded by constellation moduation per symbol is $(\log_2 C)/L + \log_2 M$ bits, where for 4D-BPSK M=2, C=4 and L=3.

Monte-Carlo simulations have been performed to assess the sensitivity of 4D-BPSK in comparison to advanced modulation formats. The BER versus SNR is plotted in Fig. S3c for PM-2QAM, PM-4QAM, PS-QPSK and 4D-BPSK with L = 3. We find that 4D-BPSK only requires a SNR as low as 4.25 dB to accomplish a BER of $1\cdot10^{-3}$. Of course, the higher sensitivity comes at the price of a lower information content per symbol. In fact, the BER plot of Fig. S3c considers only the SNR and disregards the difference in the number of bits each of the modulation formats can carry. Therefore, Fig. S3d plots the same BER curve as a function of SNR/bit. And indeed, it shows that 4D-BPSK modulation format is the most power-efficient modulation, with a sensitivity advantage of 0.75 dB and 1.7 dB in comparison to PS-QPSK and PM-4QAM respectively at a bit-error-ratio of $1\cdot10^{-3}$. The inset of Fig. S3c shows that the SNR/bit advantage remains for BER down to $1\cdot10^{-9}$. For BER $\geq1\cdot10^{-3}$, the optical space communication community investigates optionally code rates up to 0.5 [23-26], corresponding to pre-FEC BERs of $\sim1.4\cdot10^{-1}$ [24,

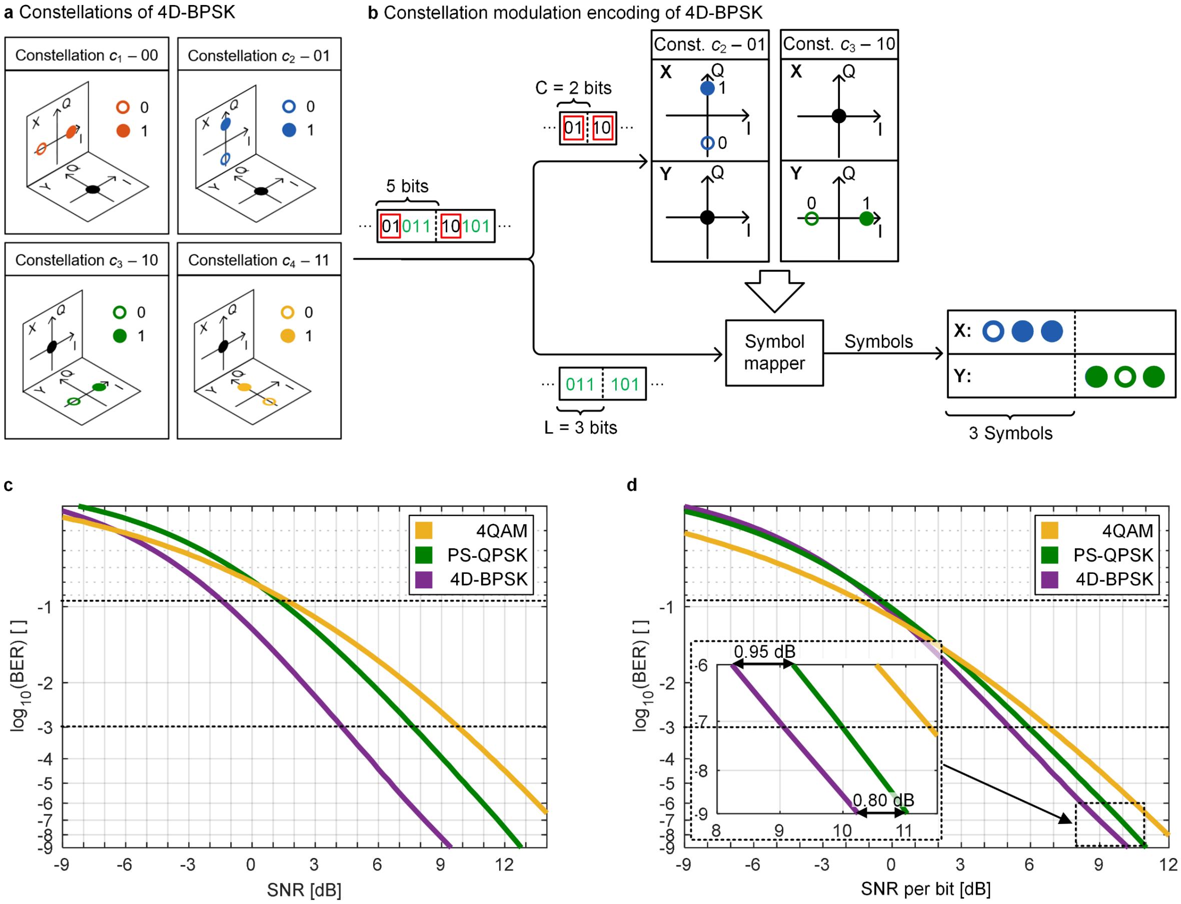

Fig. S3 Encoding a 4D-BPSK modulation format and BER versus SNR plots. a The 4D-BPSK modulation format encodes information by choosing any of 4 possible BPSK constellations (constellation modulation). There are two possible choices for encoding of BPSK in the x-polarization (constellation c_1 and c_2) and 2 possible constellations in the y-polarization (Constellation c_3 and c_4). Choosing any one of the four constellations is worth 2 bits of information. **b** Schematic representation of the constellation modulation encoder for 4D-BPSK. Here, 5 bits will be mapped onto 3 symbols. By the first two bits one out of the $C=4$ constellations is selected. This constellation is then maintained during the encoding of the next L=3 subsequent BPSK bits. So in summary, 5 bits of information are encoded by the 3 BSPK symbols. It is important to see that the high sensitivity of BPSK is maintained while in fact more information is encoded as a symbol but also through the choice of the constellation. **c** Signal-to-noise ratio (SNR) and **d** SNR per bit for sensitive modulation formats in a Monte-Carlo Simulation. In either case, the 4D-BPSK provides the best BER for the lowest SNR – except for very high BERs.

27]. Looking at Fig. S3d, we observe that curves of QPSK and 4D-BPSK cross at a BER of $6.5 \cdot 10^{-2}$. As a result, 4QAM performs better for code rates ≤ ~0.6 [24], whereas 4D-BPSK seems to be the better candidate for code rates ≥ ~0.6. Yet, FEC coding schemes add complexity and latency to the system and its implementation can be costly. Therefore, 4D-BPSK is of interest when the overhead and the FEC effort is to be minimized while a high bandwidth efficiency is of interest. This seems to be attractive for high-throughput satellite links.

Investigation of the Atmospheric Turbulence

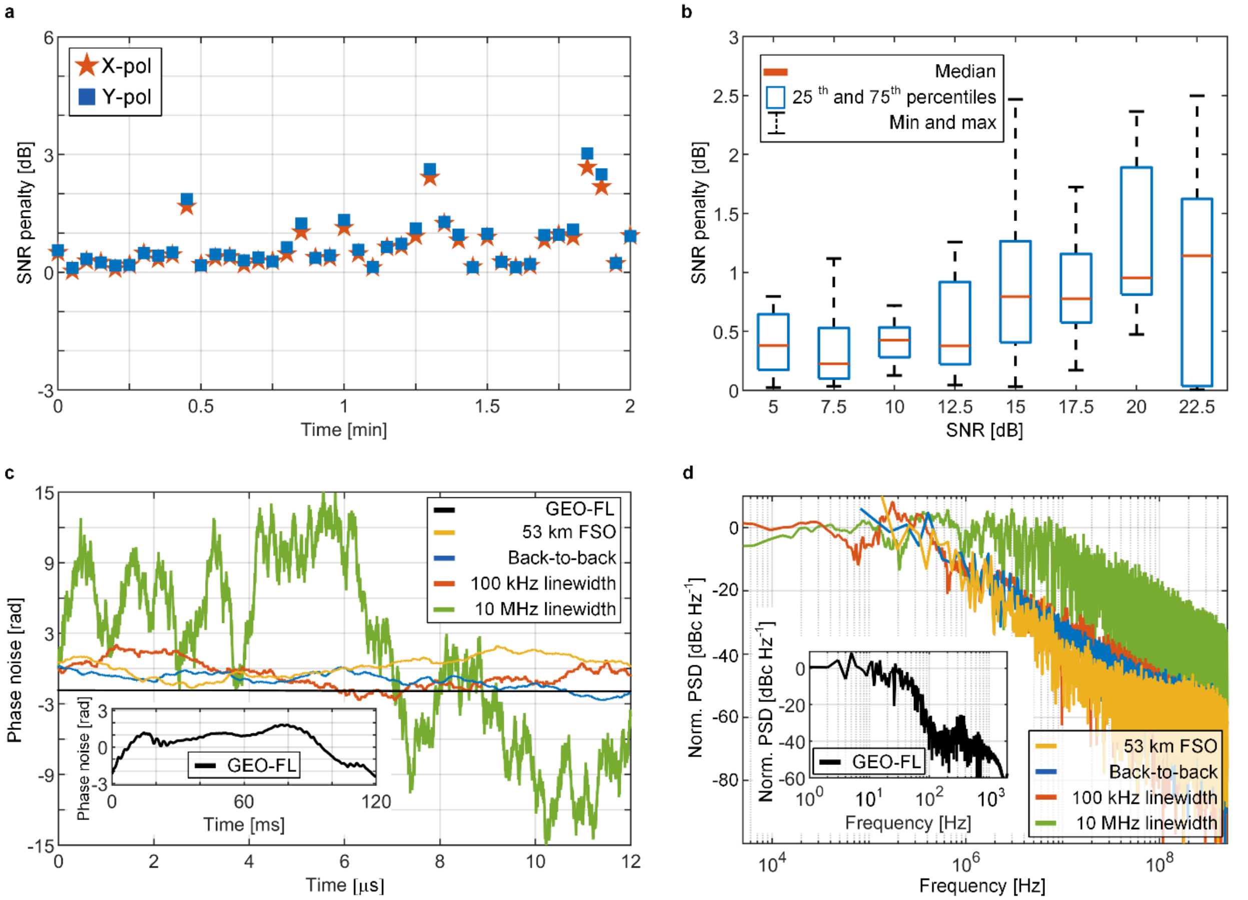

Fig. S4 Polarization Stability of the FS channel over time. a Measured SNR penalty of a static polarization demultiplexing (PMD) versus an adaptive PMD filter as a function of time. **b** Box plot of the SNR penalty as a function of the SNR. It shows that the SNR penalty slightly increases for high SNR values. The red line is the median, black dashed line shows minimum and maximum of the measurement data, and the blue box represents the range of the first and the third quartile. Estimated phase noise of the carrier phase recovery algorithm as a function of **c** time and **d** frequency. Additionally, the simulated laser phase noise for a laser linewidth of 100 kHz (red) and 10 MHz (green) is shown. The black curve corresponds to the simulated residual phase noise after FAO pre-compensation in a worst-case GEO feeder-uplink (FL), see methods section.

In fibre-optic transmission links, the receiver uses polarization demultiplexing (PDM) algorithms to compensate for the variations of the polarization state and thus, demultiplex the data back into their original states. Depending on the channel properties, the filter coefficients of PDM algorithm need to be adapted fast (in the order of milliseconds). Studies of the Gaussian Schell model beam found that the degree of polarization can slightly increase along the propagation, while the state of polarization does not vary over time in a turbulent atmosphere [28, 29]. We are interested in a quantization of this FS channel property in a high-speed data channel. Therefore, we investigate the performance of the DSP for a static versus an adaptive 2x2 PMD filtering. While for a static filter the same 2x2 FIR filter is used for all measurements, for an adaptive filter the filter coefficients are trained for each individual measurement. Fig. S4a shows the resulting SNR penalty of the static filter in comparison to an adaptive filter on a minute time scale for a 25 GBd-4D-BPSK signal. We find that the penalty in most cases stays below 1 dB. Merely for high SNR values, the median penalty gets higher by 1 dB, see Fig. S4b. This can be explained by the fact that a small error/inaccuracy of the FIR filter has bigger impact on a signal when the quality is already high.

In [30] it was shown that the residual phase noise after adaptive optics correction varies over a few rad on a few tens of milliseconds. Therefore, it would be interesting to see if the phase noise of the FS-span adds up to the laser phase noise of the optical coherent transmission system. For this, we plot the estimated phase noise of the carrier phase recovery algorithm as a function of time, see Fig. S4c, and frequency, see Fig. S4d. Here, we investigate the phase noise of a 32 GBd-PM-4QAM signal with and without the 53.42 km FS channel at an ROP of -30 dBm. Furthermore, we also simulate the laser phase noise for a laser linewidth of 100 kHz (red) and 10 MHz (green). The phase noise of a laser can be modelled as a Wiener process [31, 32]. Comparing our experiments to the simulations, we find that the laser phase noise dominates the phase noise term. In comparison to the FS phase noise, it changes in the same order of magnitude but much faster, approximately three to four orders of magnitude. Our findings align with those in

reference [28], which indicate that the polarization states of the optical signal are better preserved over time as compared to the amplitude and phase of the signal.

Measurement of Atmospheric Turbulence

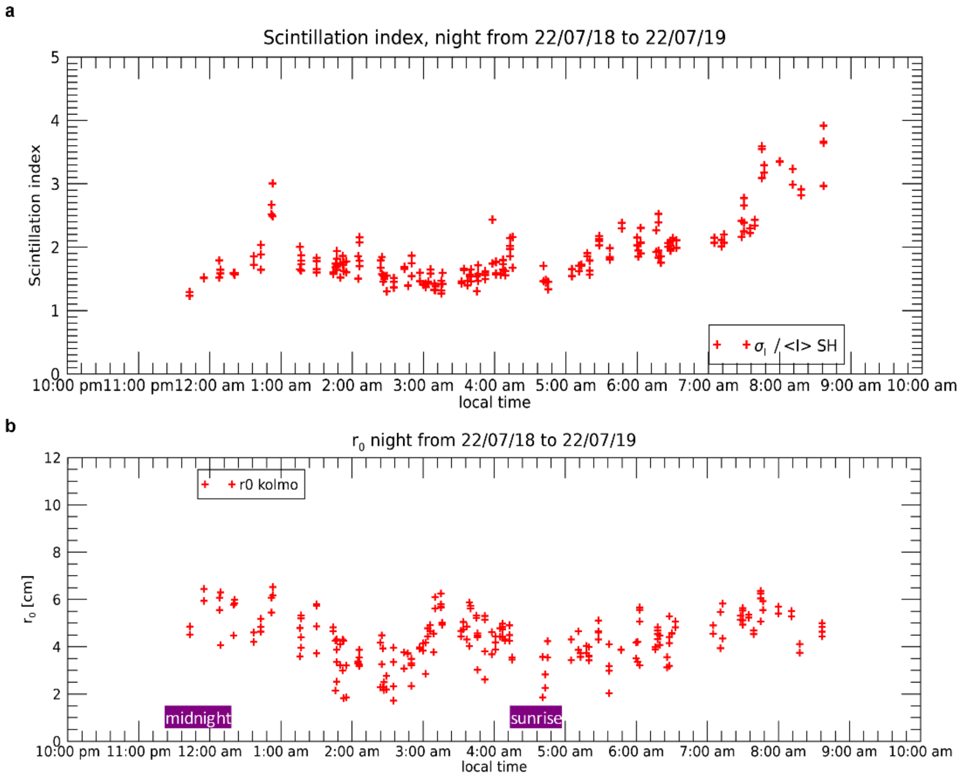

Fig. S5. Development of **a** Scintillation Index (*SI*) and **b** Fried coherence length r_0 over time. *SI* accounts for amplitude variations in the turbulent 53 km FS channel, while $r_0$characterizes for phase variations (see Main article section Results).

Experimental Setup

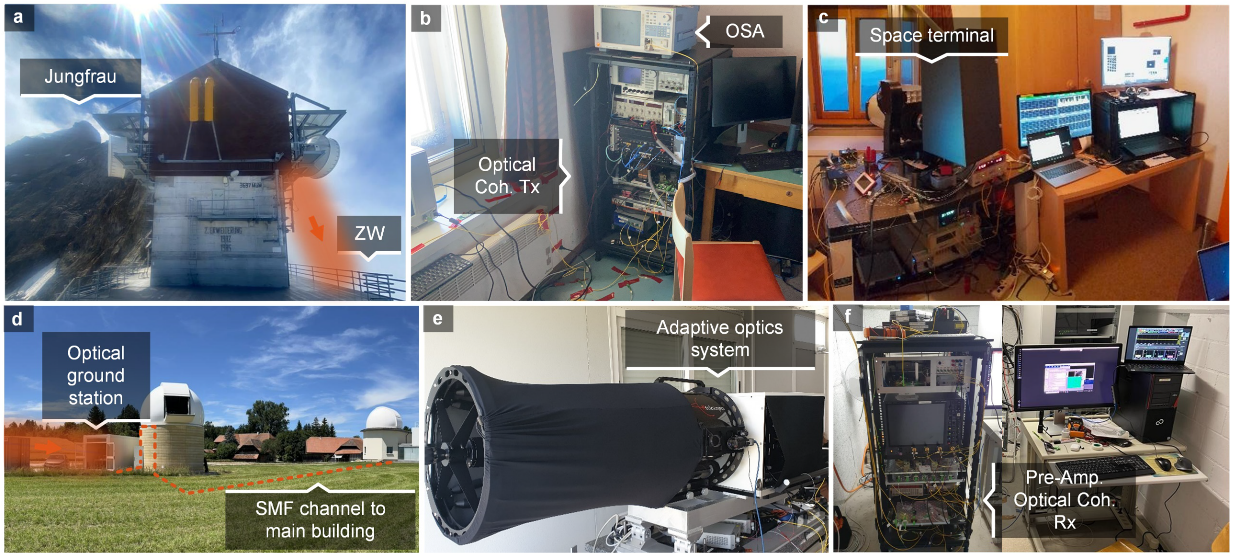

Fig. S6 Experimental Setup of the 53.4 km FSO transmission link. a-c Optical transmitter is located at the Jungfrau East Ridge and **d-f** the optical receiver situated in the Zimmerwald Observatory of University of Bern; **a** Front view of the Jungfrau East Ridge building with its side facing north-west in direction of Zimmerwald. The optical data signal propagates after generation in the **b** coherent optical transmitter through a few meters single-mode fibre and is after amplification in the HPOA emitted into free-space by the **c** telescope of the space terminal. **d** Zimmerwald Observatory where the optical ground station was placed inside of a shipping container. The optical beam is received by **e** the 35 cm telescope of the adaptive optics system and after correction coupled to a single-mode fibre. After a ~100 m fibre-span connecting the OGS to the main building, the optical signal is fed to the **f** pre-amplified optical coherent receiver (Pre-Amp. Optical Coh. Rx).

References

[1] J. Greco, *Design of the high-speed framing, FEC, and interleaving hardware used in a 5.4km free-space optical communication experiment* (SPIE Optical Engineering + Applications). SPIE, 2009.

[2] R. Lange, B. Smutny, B. Wandernoth, R. Czichy, and D. Giggenbach, *142 km, 5.625 Gbps free-space optical link based on homodyne BPSK modulation* (Lasers and Applications in Science and Engineering). SPIE, 2006.

[3] J. Juarez, D. Young, J. Sluz, J. Riggins, and D. Hughes, *Free-space optical channel propagation tests over a 147-km link* (SPIE Defense, Security, and Sensing). SPIE, 2011.

[4] N. Perlot, P. Hanne, and J. Perdigues, "Bidirectional 20-Gbit/s OOK Link with a 4-Telescope Array and Incoherent Signal Combining," in *2019 IEEE International Conference on Space Optical Systems and Applications (ICSOS)*, 2019, pp. 1-4.

[5] J. Surof, J. Poliak, and R. M. J. O. l. Calvo, "Demonstration of intradyne BPSK optical free-space transmission in representative atmospheric turbulence conditions for geostationary uplink channel," vol. 42, no. 11, pp. 2173-2176, 2017.

[6] X. Feng, Z. Wu, T. Wang, P. Zhang, X. Li, H. Jiang, Y. Su, H. He, X. Wang, and S. J. O. C. Gao, "Experimental demonstration of bidirectional up to 40 Gbit/s QPSK coherent free-space optical communication link over∼ 1 km," vol. 410, pp. 674-679, 2018.

[7] P. Conroy, J. Surof, J. Poliak, and R. Mata Calvo, "Demonstration of 40 GBaud intradyne transmission through worst-case atmospheric turbulence conditions for geostationary satellite uplink," *Applied Optics,* vol. 57, no. 18, pp. 5095-5101, 2018/06/20 2018.

[8] J. Poliak, R. M. Calvo, and F. Rein, "Demonstration of 1.72 Tbit/s Optical Data Transmission Under Worst-Case Turbulence Conditions for Ground-to-Geostationary Satellite Communications," *IEEE Communications Letters,* vol. 22, no. 9, pp. 1818-1821, 2018.

[9] A. Lorences-Riesgo, F. P. Guiomar, A. N. Sousa, A. L. Teixeira, N. J. Muga, M. C. R. Medeiros, and P. P. Monteiro, "200 G Outdoor Free-Space-Optics Link Using a Single-Photodiode Receiver," *Journal of Lightwave Technology,* vol. 38, no. 2, pp. 394-400, 2020/01/15 2020.

[10] G. Parca, A. Shahpari, V. Carrozzo, G. Beleffi, and A. Teixeira, "Optical wireless transmission at 1.6-Tbit/s (16×100 Gbit/s) for next-generation convergent urban infrastructures," vol. 52 %J Optical Engineering, no. 11, p. 116102, 2013.

[11] H.-W. Wu, H.-H. Lu, W.-S. Tsai, Y.-C. Huang, J.-Y. Xie, Q.-P. Huang, and S.-C. Tu, "A 448-Gb/s PAM4 FSO communication with polarization-multiplexing injection-locked VCSELs through 600 m free-space link," *IEEE Access,* vol. 8, pp. 28859-28866, 2020.

[12] A. Dochhan, J. Poliak, J. Surof, M. Richerzhagen, H. F. Kelemu, and R. M. Calvo, "13.16 Tbit/s Free-space Optical Transmission over 10.45 km for Geostationary Satellite Feeder-links," in *Photonic Networks; 20th ITG-Symposium*, 2019, pp. 1-3.

[13] F. P. Guiomar, A. Lorences-Riesgo, D. Ranzal, F. Rocco, A. N. Sousa, M. A. Fernandes, B. T. Brandão, A. Carena, A. L. Teixeira, M. C. R. Medeiros, and P. P. Monteiro, "Adaptive Probabilistic Shaped Modulation for High-Capacity Free-Space Optical Links," *Journal of Lightwave Technology,* vol. 38, no. 23, pp. 6529-6541, 2020.

[14] K. Matsuda, M. Binkai, S. Koshikawa, T. Yoshida, H. Sano, Y. Konishi, and N. Suzuki, "Demonstration of a Real-Time 14 Tb/s Multi-Aperture Transmit Single-Aperture Receive FSO System With Class 1 Eye-Safe Transmit Intensity," *Journal of Lightwave Technology,* vol. 40, no. 5, pp. 1494-1501, 2022.

[15] F. P. Guiomar, M. A. Fernandes, J. L. Nascimento, V. Rodrigues, and P. P. Monteiro, "Coherent Free-Space Optical Communications: Opportunities and Challenges," *Journal of Lightwave Technology,* vol. 40, no. 10, pp. 3173-3186, 2022.

[16] M. A. Fernandes, P. P. Monteiro, and F. P. Guiomar, "Single-Wavelength Terabit FSO Channel for Datacenter Interconnects Enabled by Adaptive PCS," in *2021 Optical Fiber Communications Conference and Exhibition (OFC)*, 2021, pp. 1-3.

[17] M. Karlsson and E. Agrell, "Which is the most power-efficient modulation format in optical links?," *Optics Express,* vol. 17, no. 13, pp. 10814-10819, 2009/06/22 2009.

[18] F. Pittalà, M. Schaedler, G. Khanna, S. Calabrò, M. Kuschnerov, C. Xie, Z. Ye, Q. Wang, and B. Zheng, "220 GBaud Signal Generation Enabled by a Two-channel 256 GSa/s Arbitrary Waveform Generator and Advanced DSP," in *2020 European Conference on Optical Communications (ECOC)*, 2020, pp. 1-4: IEEE.

[19] D. Moor and J. Leuthold, "Plasmonics–Key Technology to the Terabaud Age," in *8th International Symposium for Optical Interconnect in Data Centres (ECOC 2022)*, 2022.

[20] C. Fuchs and C. Schmidt, *Update on DLR's OSIRIS program* (International Conference on Space Optics - ICSO 2018). SPIE, 2019.

[21] P. Hyvönen, A. Vidmark, L. Francou, and G. Baister, "On-board terminal developments and operations of optical ground networks for small satellites," in *2018 SpaceOps Conference*, 2018, p. 2477.

[22] A. Carrasco-Casado, H. Takenaka, D. Kolev, Y. Munemasa, H. Kunimori, K. Suzuki, T. Fuse, T. Kubo-Oka, M. Akioka, Y. Koyama, and M. Toyoshima, "LEO-to-ground optical communications using SOTA (Small Optical TrAnsponder) – Payload verification results and experiments on space quantum communications," *Acta Astronautica,* vol. 139, pp. 377-384, 2017/10/01/ 2017.

[23] D. R. Arrieta, S. Almonacil, J.-M. Conan, L. Paillier, V. Michau, E. Dutisseuil, S. Bigo, J. Renaudier, and R. Boddeda, "Block Interleaver Dimensioning and Real-Time Demonstration for Ground-to-Satellite Optical Communications," in *European Conference on Optical Communication (ECOC) 2022*, Basel, 2022, p. Tu3F.2: Optica Publishing Group.

[24] E. ETSI, "302 307-1 V1. 4.1 (2014-11):"Digital Video Broadcasting (DVB-S2)," *Second generation framing structure, channel coding and modulation systems for Broadcasting, Interactive Services, News Gathering and other broadband satellite Applications*.

[25] M. L. Stevens, D. O. Caplan, B. S. Robinson, D. M. Boroson, and A. L. Kachelmyer, "Optical homodyne PSK demonstration of 1.5 photons per bit at 156 Mbps with rate-½ turbo coding," *Optics Express,* vol. 16, no. 14, pp. 10412-10420, 2008/07/07 2008.

[26] M. B. Don, S. R. Bryan, V. M. Daniel, A. B. Dennis, K. Farzana, M. K. Joseph, S. Zoran, and M. C. Donald, "Overview and results of the Lunar Laser Communication Demonstration," in *Proc.SPIE*, 2014, vol. 8971, p. 89710S.

[27] R. Kakarla, M. Mazur, J. Schröder, and P. A. Andrekson, "Power Efficient Communications Employing Phase Sensitive Pre-Amplified Receiver," *IEEE Photonics Technology Letters,* vol. 34, no. 1, pp. 3-6, 2022.

[28] X. Zhao, Y. Yao, Y. Sun, and C. Liu, "Circle Polarization Shift Keying With Direct Detection for Free-Space Optical Communication," *Journal of Optical Communications and Networking,* vol. 1, no. 4, pp. 307-312, 2009/09/01 2009.

[29] X. Tang, Z. Ghassemlooy, S. Rajbhandari, W. O. Popoola, and C. G. Lee, "Coherent Heterodyne Multilevel Polarization Shift Keying With Spatial Diversity in a Free-Space Optical Turbulence Channel," *Journal of Lightwave Technology,* vol. 30, no. 16, pp. 2689-2695, 2012.

[30] C. Robert, J.-M. Conan, and P. Wolf, "Impact of turbulence on high-precision ground-satellite frequency transfer with two-way coherent optical links," *Physical Review A,* vol. 93, no. 3, p. 033860, 03/31/ 2016.

[31] E. Ip and J. M. Kahn, "Feedforward Carrier Recovery for Coherent Optical Communications," *Journal of Lightwave Technology,* vol. 25, no. 9, pp. 2675-2692, 2007.

[32] J. R. Barry and E. A. Lee, "Performance of coherent optical receivers," *Proceedings of the IEEE,* vol. 78, no. 8, pp. 1369-1394, 1990.
